# A novel proteomics approach to epigenetic profiling of circulating nucleosomes

**DOI:** 10.1038/s41598-021-86630-3

**Published:** 2021-03-31

**Authors:** Priscilla Van den Ackerveken, Alison Lobbens, Jean-Valery Turatsinze, Victor Solis-Mezarino, Moritz Völker-Albert, Axel Imhof, Marielle Herzog

**Affiliations:** 1 Belgian Volition SRL, 22 Rue Phocas Lejeune, Parc Scientifique Crealys, 5032 Isnes, Belgium; 2 EpiQMAx GmbH, Am Klopferspitz 19, 82152 Planegg-Martinsried, Germany

**Keywords:** Biomarkers, Colorectal cancer, Histone analysis, Immunoprecipitation, Mass spectrometry, Proteomic analysis

## Abstract

Alteration of epigenetic modifications plays an important role in human cancer. Notably, the dysregulation of histone post-translational modifications (PTMs) has been associated with several cancers including colorectal cancer (CRC). However, the signature of histone PTMs on circulating nucleosomes is still not well described. We have developed a fast and robust enrichment method to isolate circulating nucleosomes from plasma for further downstream proteomic analysis. This method enabled us to quantify the global alterations of histone PTMs from 9 CRC patients and 9 healthy donors. Among 54 histone proteoforms identified and quantified in plasma samples, 13 histone PTMs were distinctive in CRC. Notably, methylation of histone H3K9 and H3K27, acetylation of histone H3 and citrullination of histone H2A1R3 were upregulated in plasma of CRC patients. A comparative analysis of paired samples identified 3 common histone PTMs in plasma and tumor tissue including the methylation and acetylation state of lysine 27 of histone H3. Moreover, we highlight for the first time that histone H2A1R3 citrulline is a modification upregulated in CRC patients. This new method presented herein allows the detection and quantification of histone variants and histone PTMs from circulating nucleosomes in plasma samples and could be used for biomarker discovery of cancer.

## Introduction

Colorectal cancer (CRC) is the 4th most common cancer in the world^1^. CRC results from an accumulation of genetic and epigenetic alterations in colonic epithelial cells that transforms them into adenocarcinomas^2,3^. Despite improvements in diagnosis and screening strategies, the survival rate for advanced cancer is still low. However, it could be improved with early diagnosis, because mortality decreases if the cancer is identified at early stages when treatment is more effective. Epigenetic biomarkers are emerging as tools for the early detection of various diseases including cancer^4^. In the nucleus of eukaryotic cells, DNA is wrapped around eight histone proteins to form the fundamental repeating unit of chromatin called the nucleosome. Each nucleosome octamer is composed by two copies of each core histone protein: H2A, H2B, H3 and H4. In addition, all core histones could also have a variety of post-translational modifications (PTMs) involved in the modulation of chromatin accessibility and thereby in the regulation of key processes like transcription, cell differentiation and cell death^5,6^. An increased number of nucleosomes is detected in the blood of cancer patients^7–10^. The origin of nucleosomes in blood is still unclear. However, in several type of cancers, the release of circulating nucleosomes has been associated with tumor burden and malignant progression^11,12^. In addition, it has been demonstrated that genome-wide epigenetic signals are altered in cancer and accumulating evidence indicates that epigenetic changes occur early in tumorigenesis^13^. For example, dysregulation of histone modifications has been associated with CRC or pancreatic cancer suggesting that changes in histone modification patterns detected on circulating nucleosomes could therefore be powerful blood-based biomarkers enabling early cancer detection^14–16^.

Nevertheless, tools to investigate candidate epigenetic biomarkers directly from patient blood for diagnosis or for patient stratification are limited^17^. While immunoassays could provide a reliable quantitative measure of circulating nucleosomes and their associated epigenetic modifications, it is necessary to already know which histone PTMs are potentially relevant modifications. Mass spectrometry is a powerful solution for biomarker discovery. Currently, most of blood proteomic work has been conducted to analyze total protein abundance, with very few studies to analyze specifically circulating nucleosomes/histones^18^. Mass spectrometry studies on histones PTMs have been mostly conducted on cell lines or tissues^19–21^. Indeed, one challenge to study histones PTMs in blood is the matrix complexity where the high protein content and the abundance of blood proteins may mask histones PTMs. In this context, it is critical to develop a method to enrich blood samples in circulating nucleosomes to study their associated histones PTMs.

In this study, we present a new method for the detection and the quantification of circulating nucleosomes and their associated histone epigenetic modifications in plasma samples. This method is based on the capture of intact circulating nucleosomes using a newly developed immunoprecipitation method followed by liquid chromatography coupled with tandem mass spectrometry (Nu.Q Capture-MS). To identify histone PTM signatures of circulating nucleosomes and, subsequentially, discover new CRC biomarkers, we implemented this innovative method in a pilot study composed by plasma samples from cancer patients and healthy controls. We identified the highest level of H3.1-positive nucleosome in cancer patients and a pattern of 13 histone PTMs differentially represented in plasma from cancer patients compared to healthy controls. Moreover, the comparison between the epigenetic profile found on circulating nucleosomes and the pattern detected in the tumor tissue from the same patient indicate that three of them are reflecting the epigenetic signature of the tumor. Therefore, the developed method Nu.Q Capture-MS is a powerful new technique, allowing the quantification of histone PTMs in plasma samples and the discovery of potential biomarkers for cancer diagnosis.

## Results

### Nu.Q Capture enriches for H3.1 circulating nucleosomes from plasma samples

**Figure 1:**
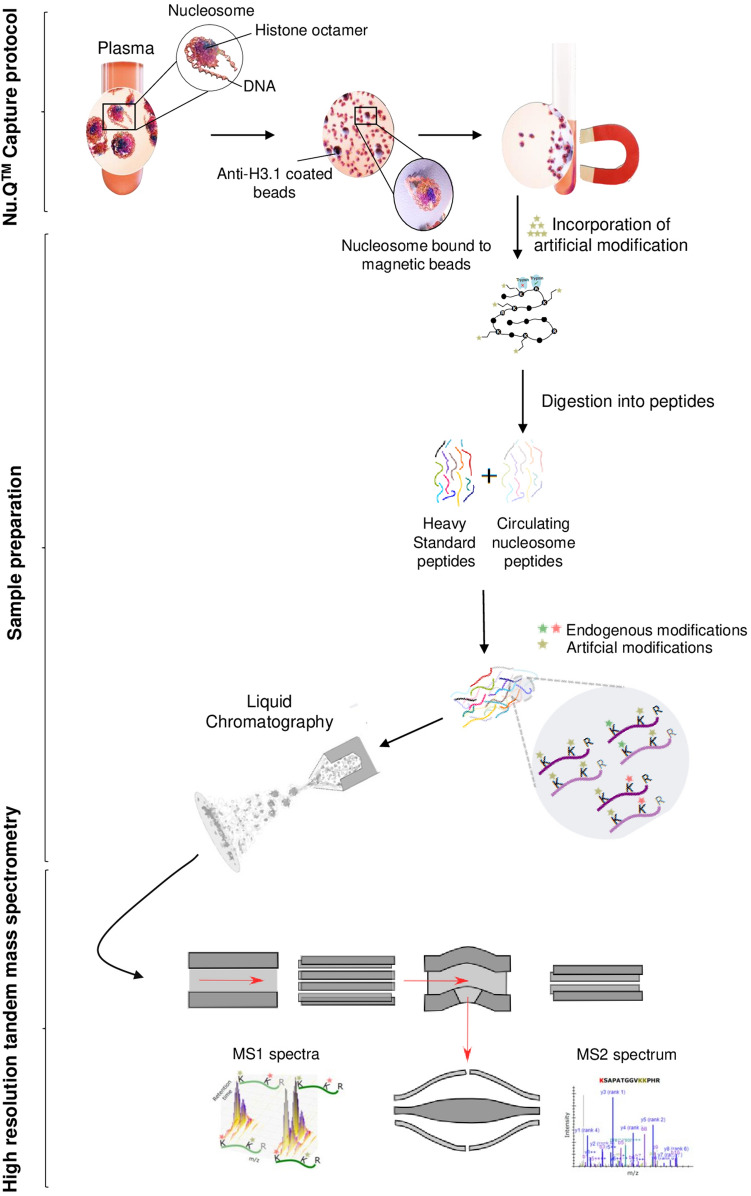
Workflow of the Nu.Q Capture—Mass spectrometry process. 900 µl of Plasma samples containing circulating nucleosomes were incubated with anti-H3.1 coated magnetic beads (Nu.Q Capture protocol) to isolate, nucleosomes captured from the rest of plasma. Then, chemical derivatization of histones by acylation (here referred as “incorporation of artificial modification”) was used to block the lysine residues and generate compatible peptides (here referred as circulating nucleosome peptides) for LC-MS analysis. After trypsin digestion, heavy amino acid-labeled histone H3 peptides (here referred as heavy standard peptides) were added during sample preparation to each sample. These synthetic histone peptides are used for normalization to eliminate potential bias caused by sample preparation or instrumentation. Next, desalted peptides were injected in a liquid chromatography system (Ultimate 3000 RSLCnano). The effluent from the HPLC was directly electro-sprayed into a Q Exactive HF mass spectrometer (Thermo Fisher Scientific, San Jose, CA). The mass spectrometer was operated in MS/MS acquisition mode.

**Figure 2:**
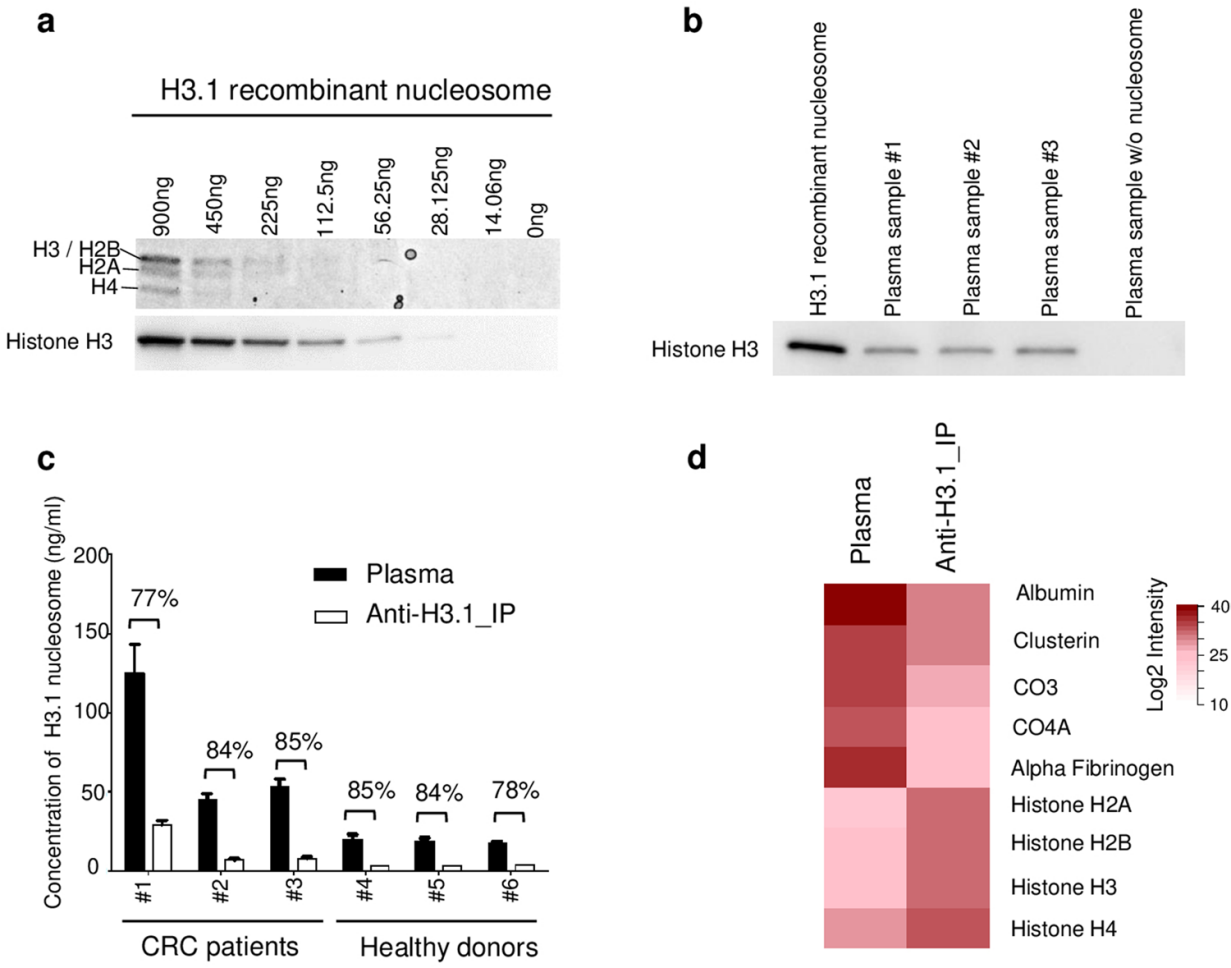
Histone H3.1 immunoprecipitation to enrich blood samples in circulating nucleosomes. (**a**) Coomassie blue staining and anti-histone H3 Western blot analyses on the immunoprecipitated material (cropped gel/blot pictures; full-length gel/blot are presented in Supplementary Fig. 1). (**b**) Western blot analysis using an anti-histone H3 (mAb) on the immunoprecipitated material from H3.1 recombinant nucleosome (positive control) or plasma samples from CRC patients (n = 3 labelled from #1 to #3) or a plasma sample without detectable level of circulating nucleosomes (negative control). (**c**) Nu.Q H3.1 ELISA results showing the depletion of nucleosomes after Nu.Q Capture immunoprecipitation (anti-H3.1_IP) in comparison to the level present in the initial plasma (plasma) (All data are presented as mean ± SD). The percentage of depletion was indicated for all samples (n = 3 healthy donors and n = 3 CRC). The concentration of circulating nucleosome was determined by interpolating the mean value of each tested sample against a standard curve made of recombinant nucleosomes. Results are expressed in ng/mL. (**d**) Intensity of all proteotypic peptides of the mentioned proteins. Representative blood proteins peptides and histone proteins detected by mass spectrometry in unprocessed plasma samples or after nucleosomes enrichment with the Nu.Q Capture protocol (anti-H3.1_IP). The average intensity of the 2 technical replicates in each experimental group was calculated peptide-sequence-wise (Listed in Supplementary Table 1). Next, the average intensity of all peptides was summarized protein-ID-wise. After this, average intensity values were log2-transformed and plotted as a red scale heatmap.

The abundant concentration of blood proteins such as albumin, fibrinogen, apolipoproteins or complement factors prevent the detection of lower-abundance proteins like circulating nucleosomes and histone PTMs by mass spectrometry. To allow the proteomic analysis of PTMs present on circulating nucleosomes, we developed an enrichment method based on nucleosome immunoprecipitation (Nu.Q Capture) (Fig. 1). 900 µl of K2-EDTA plasma samples were incubated with anti-histone H3.1 antibody coated on magnetic particles. To confirm that the Nu.Q Capture protocol allows immunoprecipitation of nucleosomes from plasma samples in a quantitative manner, we either spiked defined quantities of recombinant H3.1 nucleosome into a plasma matrix containing no nucleosomes or tested native plasma samples. Then, we assessed the pull-down efficacy by Coomassie blue staining and anti-histone H3 Western blot analysis on the immunoprecipitated material. As expected, we observed a dose-dependent signal for all histone cores as well as for histone H3 and we confirmed the immunoprecipitation of nucleosomes in native samples (Fig. 2a,b and Supplementary Fig. 1). Hence, the quantity of the immunoprecipitated nucleosome is proportional to the initial material and subsequently allows proteomic analysis reflecting the quantity of circulating nucleosomes present in the plasma from patients. Furthermore, we wanted to assess the immunoprecipitation efficacy by verifying the depletion of nucleosomes in clinical samples after the Nu.Q Capture protocol. For this purpose, we performed Nu.Q-H3.1 ELISA to quantify the levels of nucleosomes in plasma samples from CRC patients and heathy donors. As described, we noted in the starting material that the level of circulating nucleosomes is higher in CRC patients compared to healthy donors (Fig. 2c). Then, the concentration of circulating nucleosomes remaining in plasma after the immunoprecipitation step was compared to the normal level present in unprocessed plasma samples. Nucleosome quantification showed a mean depletion of 82.2% ± 3.7% (n = 3 healthy donors and n = 3 CRC patients) after immunoprecipitation confirming the pull-down efficacy of the method.

To examine whether this newly developed method allows the nucleosome component analysis by mass spectrometry, plasma sample from the same patient with or without nucleosome immunoprecipitation was subjected to LC-MS/MS. Without immunoprecipitation, 95% of the detected peptides were from common blood proteins whereas histone peptides were almost undetectable (Fig. 2d and Supplementary Table 1). In contrast, due to the nucleosome enrichment method, we observed an increase of histone peptide detection leading to a sequence coverage > 60% and a drastic reduction of peptides associated with blood proteins by 52% (Fig. 2d and data not shown). Altogether these data suggest that the Nu.Q Capture protocol is effective to isolate circulating nucleosomes from plasma samples to allow their subsequent analysis by mass spectrometry.

### Nu.Q Capture-MS allows the epigenetic profile analysis of circulating nucleosomes

**Figure 3:**
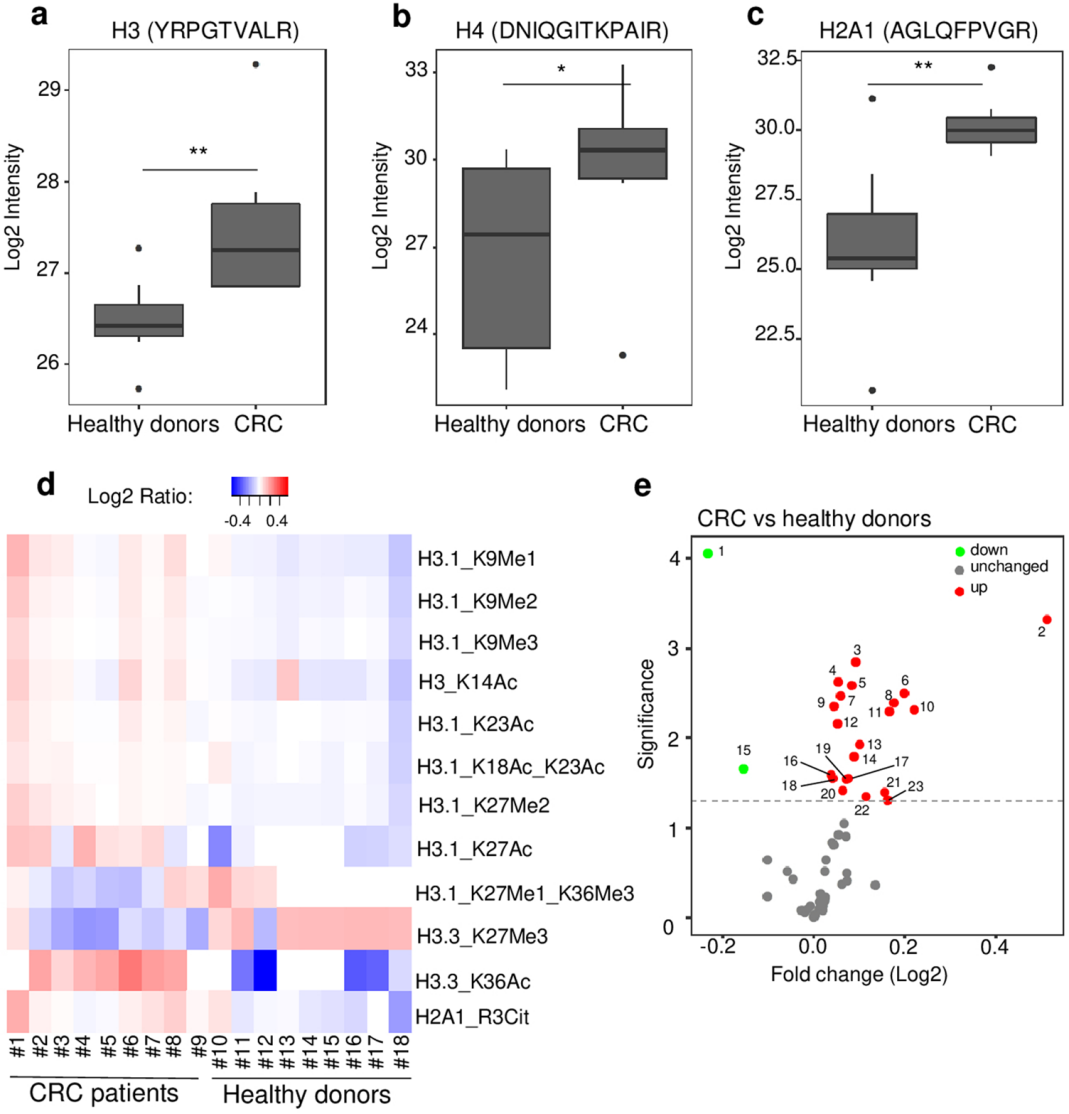
Nu.Q Capture-MS allows the analysis of histone PTMs from circulating nucleosomes. Box plot showing the abundance of unmodifiable histone peptides H3 (**a**), H4 (**b**), H2A1 (**c**) detected by Nu.Q capture-MS in plasma sample from healthy donors (n = 9) or CRC patients (n = 9). *p*-values were determined by student’s t- Test (*p < 0.05; **p < 0.01). The box plot shows the median and the 25th and 75th percentiles; the whiskers indicate the 5th and 95th percentiles. CRC patients (#1 to #9) and healthy donors (#10 to #18) were indicated by different sample numbers. (**d**) Heat map showing the histone PTMs peptides (listed in Supplementary Table 2) differentially abundant between CRC patients (n = 9) and healthy donors (n = 9); p < 0.05. Data are shown as Log2 ratio of histone PTM levels (log 2 intensity) for a given sample based on average PTM levels across all samples. Log 2 intensity refers to heavy peptide normalized intensity. (**e**) Volcano plot representing the significance (expressed as −log10(*p*-value)) as a function of the magnitude of fold change between CRC patients (n = 9) vs healthy donors (n = 9) of the 54 histone proteoforms identified in plasma using the Nu.Q Capture-MS. 23 were significantly dysregulated including 2 downregulated (green) and 21 upregulated (red) (*p* < 0.05). Each plot with a significant fold change is labelled and recorded in Supplementary Table 3A.

To discover and quantify epigenetic histone modifications present in plasma samples, we applied the newly developed Nu.Q Capture-MS protocol in a pilot study composed by plasma samples from CRC patients (n = 9) and healthy donors (n = 9). After nucleosome enrichment utilizing the Nu.Q-Capture protocol and digestion into peptides, the plasma samples were analyzed by LC-MS/MS. The quantification of the peptides was achieved by using isotope labelled spike-in standards, which consist of heavy labeled modified or unmodified histones peptides that are introduced to the processed clinical plasma sample and are subjected to the same processing steps, to control experimental variability and increase quantitation accuracy. By comparing CRC vs healthy plasma samples, 54 histone proteoforms were identified in plasma. As predicted, core histone proteins such as histone H3, H4 or H2A were identified in all immunoprecipitated samples (Supplementary Table 2). These data confirm that the anti-H3.1 immunoprecipitation protocol is effective to isolate circulating nucleosomes from plasma samples. Among the histone peptides identified, we found 23 histone proteoforms differentially represented in plasma from CRC patients or healthy donors (*p* < 0.05, Supplementary Table 2. The intensity quantification of the unmodifiable histone peptides “H3_YRPGTVALR”, “H4_ DNIQGITKPAIR” and “H2A1_AGLQFPVGR” exhibit a significant higher abundance in CRC samples compared to healthy controls (mean of log_2_ intensity = 27.46 vs 26.48 (*p* < *0.01*), 29.82 vs 26.65 (*p* < *0.05)*; *30.18 vs 25.91 (p* < *0.01)*; respectively) confirming higher levels of circulating nucleosomes in CRC patients (Fig. 3a–c). Interestingly, the identification of these 23 differentially abundant histone peptides in CRC samples allowed for the characterization of 13 distinct histone PTMs located at 7 different sites (Fig. 3d). Notably, levels of H3K9 methylation (H3K9Me1, -Me2, -Me3), H3K27 methylation (H3K27Me1, -Me2), H3 acetylation (H3K14Ac, H3K18Ac, H3K23Ac, H3K27Ac, and H3K36Ac) as well as levels of H2A1R3 citrulline (H2A1R3Cit), were significantly more elevated in CRC compared to healthy controls (Fig. 3e and Supplementary Table 3a). In contrast, we observed a significant downregulation of H3.3_K27Me3 and H3.1_K27Me1_K36Me3 modified peptides in CRC plasma in comparison to healthy donors (Fig. 3e and Supplementary Table 3a). Then, we decided to confirm the mass spectrometry results by an independent method applied on the same samples. For this purpose, we took advantage of the availability of some Nu.Q immunoassays to measure either the level of H3.1 positive nucleosomes or specific epigenetic marks on nucleosomes such as H3K9Me3, H3K27Me2/3, H3K14Ac or H3K27Ac. We have analyzed the same 9 plasma samples from CRC patients and 7 out of the 9 healthy donors by immunoassays (we run out of volume for two of them) (Supplementary Fig. 2). As expected, we observe similar results with both methods with a higher concentration of H3.1-, H3K9me3-, H3K27Me2/Me3- and H3K14Ac-nucleosomes in CRC patients compared to Healthy donors. We did not observe a significant difference in the level of H3K27Ac in CRC vs healthy. But we can’t exclude it could be due to the analytical sensitivity of this immunoassay (data not shown). Altogether, these results demonstrate that the epigenetic pattern of circulating nucleosomes can be analyzed by mass spectrometry thanks to the Nu.Q Capture protocol. Moreover, this analysis suggests that the PTM profile of circulating nucleosomes is different between CRC patients and healthy donors pointing out the potential implication of this newly developed technique for biomarker discovery and/or diagnostic purposes.

### H3K27Me2, H3K27Ac, and H2A1R3Cit histone PTMs present on circulating nucleosome reflects the epigenetic signature of the tumor

As the precise origin of circulating nucleosome remains unclear, we wanted to identify the fraction of tumor-derived nucleosomes in blood from cancer patients. To this aim, levels of histone PTMs present in plasma were compared to the levels present in solid tumor resection (hereafter referred as CRC tissues) or normal adjacent tissues (NAT) from the same patients (n = 9 paired samples; Supplementary Table 4).

**Figure 4:**
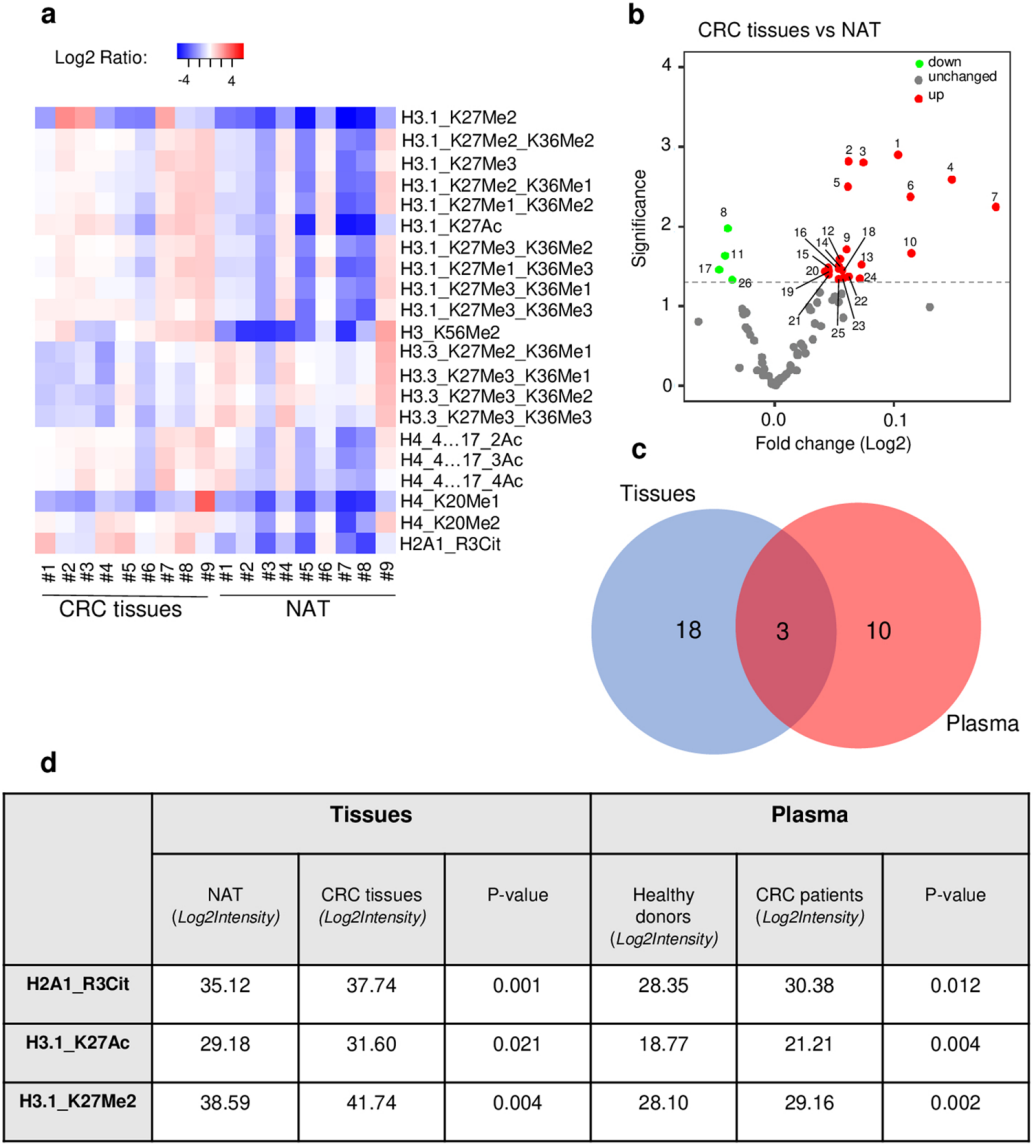
H3K27Me2, H3K27Ac, and H2A1R3Cit histone PTMs present on circulating nucleosome reflects the epigenetic signature of the tumor. (**a**) Heat map showing the histone PTMs peptides (listed in Supplementary Table 4) differentially abundant between tumor resection tissue (CRC tissues) and normal adjacent tissue (NAT) from the same patients (n = 9 paired samples; *p* < 0.05). Matched CRC and normal tissues are indicated by the same sample number (from #1 to #9). Data are shown as Log2 ratio of histone PTM levels (log 2 intensity) for a given sample based on average PTM levels across all samples. Log2 Intensity refers to heavy peptide normalized intensity. (**b**) Volcano plot representing the significance (expressed as -log10(p-value)) as a function of the magnitude of fold change between CRC tissues vs NAT (n = 9 paired samples) of the 79 histone proteoforms identified in plasma. 26 were significantly dysregulated including 4 downregulated (green) and 22 upregulated (red) (*p* < 0.05). Each plot with a significant fold change is labelled and recorded in Supplementary Table 3. (**c**) Venn diagram analysis showing the logical relation between the histone PTMs peptides differentially expressed in tumor tissues (blue) and plasma (red), highlighting the 3 common histone PTMs (Other plots are recorded in Supplementary Table 5). (**d**) Table with log2 intensity value of the 3 common histone PTMs similarly dysregulated in plasma and tumor tissue from the same patient, (n = 9 paired samples; *p* < 0.05).

Firstly, we focused our analysis on tissue samples, and we identified 79 histone proteoforms by mass spectrometry. Among them, 26 histone peptides were differentially abundant in CRC tissue vs NAT (*p* < 0.05; Supplementary Table 4. Interestingly, these peptides exhibit 21 distinctive histone PTMs located at 9 different sites (Fig. 4a, p < 0.05). Notably, levels of H3K27 methylation (H3K27Me1, -Me2, -me3), H3K36 methylation (H3K36Me1, -Me2, -Me3), H3K56 methylation (H3K56Me2), H3K27 acetylation (H3K27Ac), H4K20 methylation (H4K20Me1, -Me2; but not H4K20Me3), H4 acetylation (H4K4…17_2Ac, -3Ac, -4Ac), and H2A1R3 citrulline (H2AR13Cit) were statistically more elevated in CRC tissues compared to NAT controls (Fig. 4b, Supplementary Table 3B). Besides this increase, we also noticed a significant reduction of the methylation state of lysine 27 and 36 present on the variant H3.3 (Fig. 4b, Supplementary Table 3B). Interestingly, we did not observe a significant fold change or a modification in the total level of H3K9Me3 (given by the sum of peptides H3K9me3 and H3K9Me3_K14Ac) between the tumor and normal tissues, but we observed a significant increase of the percentage of relative abundance of the H3K9Me3 unacetylated peptide in the tumor tissue samples compared to normal samples (Data not shown).

Then, to assess whether the histone PTMs present on circulating nucleosomes reflect the epigenetic alteration present in tumor tissues, we checked if there is any logical relation between the set of histone PTMs identified in CRC tissues (21 histone PTM peptides) and the set of histone PTMs peptides significantly mis-regulated in plasma of CRC patients (13 histone PTM peptides).

As shown by the Venn diagram, this analysis highlighted 3 common histone PTMs similarly dysregulated in plasma and tumor tissue from the same patients, including the methylation and acetylation state of the lysine 27 (H3K27Ac) and the citrullination of the histone H2A1 (H2A1R3Cit) (Fig. 4c,d and Supplementary Table 5). Altogether, these data suggest that these 3 histone PTMs present on circulating nucleosome could be tumor-derived and reflect the epigenetic signature of the tumor, whereas the 10 other histone PTMs on circulating nucleosome would be more tumor-associated.

### Histone H3K27 and H3K9 PTMs identified by mass spectrometry could be potential biomarkers for the diagnosis of colorectal cancer

**Figure 5:**
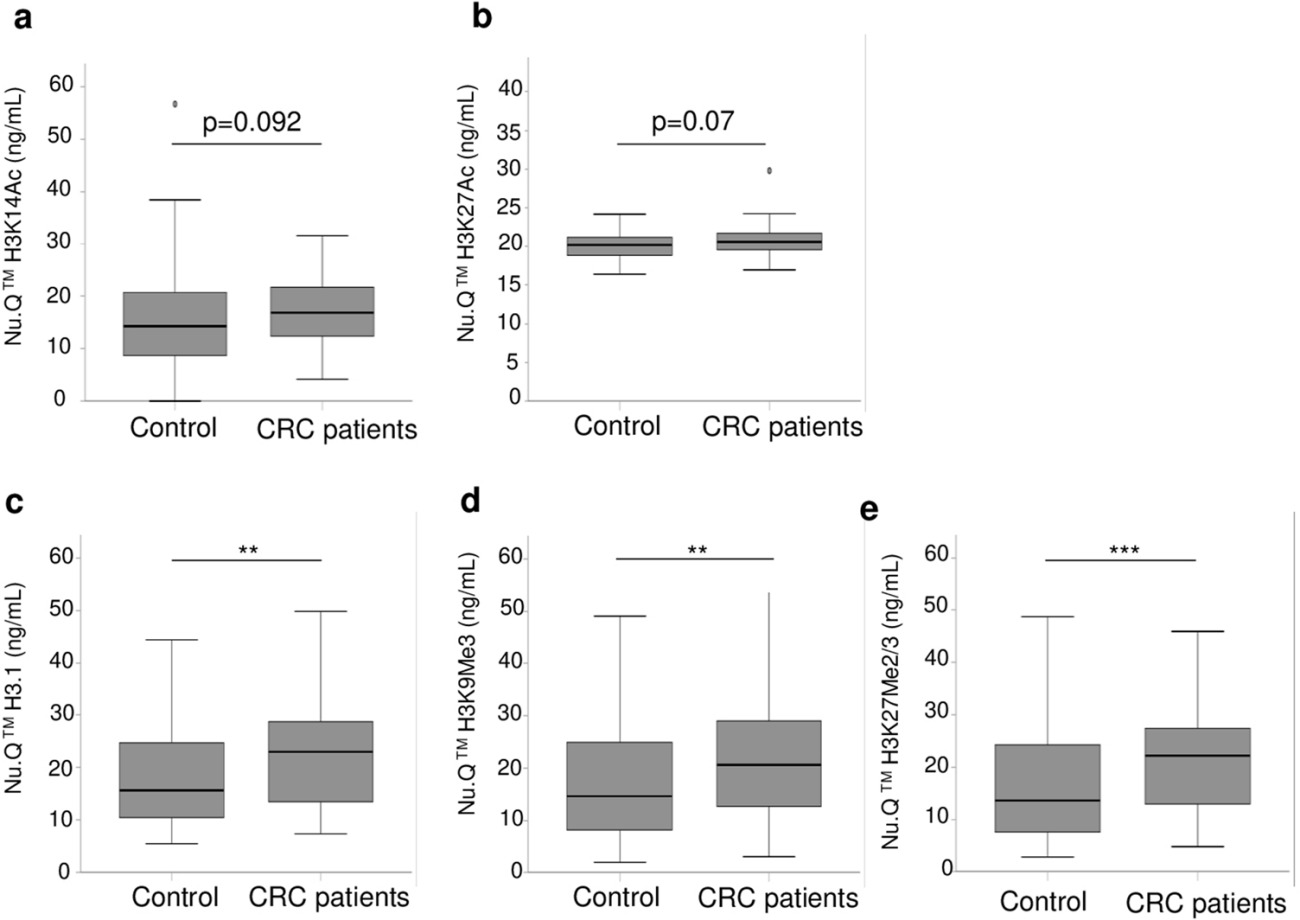
Confirmation of Nu.Q Capture-MS results by immunoassays in an independent clinical cohort. Quantification by immunoassays of circulating nucleosomes containing H3K14Acetyl (**a**) or H3K27Acetyl (**b**) H3.1 variant (**c**) H3K9Me3 (**d**) or H3K27Me2/3 (**e**) in plasma from control subjects (n = 192) and CRC patients (n = 64). The box plot shows the median and the 25th and 75th percentiles; the whiskers indicate the 5th and 95th percentiles. Significant higher levels of circulating nucleosome with H3.1, H3K9Me3 or H3K27Me2/3 but not for H3K14Ac and H3K27Ac-nucleosomes were observed in CRC patients. *p*-values were determined by Mann–Whitney (**p < 0.01 ***p < 0.001).

Having identified epigenetic marks as mis-regulated in plasma samples of CRC patients, we decided to confirm the mass spectrometry results by an independent method with a higher number of clinical samples (n = 256). For this purpose, we took advantage of the availability of some Nu.Q immunoassays to measure either the level of H3.1 positive nucleosomes or specific epigenetic marks on nucleosomes such as H3K9Me3, H3K27Me2/3, H3K14Ac or H3K27Ac. We analyzed 64 cancer patients and 192 control colonoscopy-negative subjects (hereafter referred as control) (Fig. 5). While there were no significant differences in H3K14Ac level (median 16.9 vs 14.3, *p* = 0.092; Fig. 5a) and H3K27Ac (median 20.5 vs 20.2, *p* = 0.07; Fig. 5b) between CRC and controls, significant discrimination was observed between the CRC and the control groups for nucleosomes with H3.1 (median 23 vs 15.6, *p* = 0.002*;* Fig. 5c), H3K27Me2/3 (median 22.1 vs 13.6, *p* = 0.001*;* Fig. 5e), and H3K9Me3 (median 20.6 vs 14.7, *p* = 0.004; Fig. 5d). These data confirmed that the level of circulating H3.1 positive nucleosome and the methylation state on lysine 9 and 27 are altered in colorectal cancer and could serve as potential biomarkers for colorectal cancer.

Altogether, these results demonstrated that the epigenetic pattern of circulating nucleosomes can be analyzed by mass spectrometry thanks to the Nu.Q Capture-MS protocol. Moreover, this analysis suggested that the PTM profile of circulating nucleosomes is different between CRC patients and healthy donors, pointing out the potential implication of this newly developed technique for biomarker discovery and or diagnostic purposes.

## Discussion

Human cancer cells harbor genetic alterations and an accumulation of epigenetic abnormalities. One mechanism of epigenetic regulation is the post-translational modification of histones. Any alteration in this process modifies the chromatin structure and leads to genome instability and gene misexpression. Numerous studies have highlighted that the disruption of the epigenome is a fundamental mechanism in cancer development and progression^11,13,22^. Given their key involvement in carcinogenesis, histone PTMs are emerging as potential biomarkers in clinical diagnostics and prognostics of cancer as well as therapeutic targets^23^. While most work studying histone PTMs are carried out on tumor resection or cells lines^24–26^, we propose here a new method to identify and quantify epigenetic alterations directly from the blood of patients. Using the combination of the capture of circulating nucleosomes and the underlying analysis of histone PTMs by tandem mass spectrometry, we significantly reduced the abundance of blood proteins, allowing the characterization of histone PTMs in plasma. In addition to the advantage of using a liquid biopsy technique which is minimally invasive for the patient, this method requires low volumes of blood. Here, we applied this newly developed method on a pilot cohort composed by K2-EDTA plasma from healthy donors and colorectal cancer patients. As described, the quantification of unmodifiable peptides of core histones confirmed the higher level of circulating nucleosomes in colorectal cancer blood^9,27^. This is correlated with results obtained with the circulating nucleosomes quantification using Nu.Q H3.1 immunoassay.

The bottom-up proteomic approach used to identify histone PTMs alteration highlighted 54 proteoforms of histones in blood and 79 different histone peptides in tissue. Unexpectedly, despite the H3.1 positive-nucleosome immunoprecipitation, we observed a few H3.3 proteoforms. Knowing the two variants H3.1 and H3.3 differ only by 5 amino acid residues in humans, we cannot exclude a lack of specificity of our H3.1 antibody, even if no cross-reactivity were observed by immunoassay or western blotting post-immunoprecipitation. However, it has also been described that nucleosomes circulate as mono-nucleosomes and di-, tri- or poly-nucleosomes. In this polynucleosome case, captured H3.1-nucleosomes could be isolated with the other associated nucleosomes containing H3.3 variants. Another hypothesis would be the presence of asymmetric H3.1–H3.3 nucleosome^28^. Further investigation would be required to discriminate between these hypotheses, but none of them would have an impact on the level of histone PTMs proteoforms described in this study. Nevertheless, it would be interesting in a future study to analyze the imbalance between histone H3.1 and H3.3 variants, as the expression could reflect an aberrant transcriptional state and lead to aberrant methylation status of lysine 27 or specific histone mutation as shown in glioma^29^.

The comparison between the epigenetic pattern of CRC and healthy donors of plasma samples showed a dysregulation of 13 distinct histone PTMs whereas analysis of the paired tissue samples (CRC vs NAT) showed 21 distinct histone PTMs. By comparing histone PTMs in both plasma and tissues, we found that blood plasma nucleosomes have maintained the tumor lysine K27 methylation and acetylation states as well as the H2A1R3 citrullination profile speculating that these circulating nucleosomes have a tumor origin. In contrast, alteration of the H3 lysine K9 methylation status and of the global acetylation level of histone H3 seems to be more specific to blood and not having a direct tumor origin. However, the origin of the circulating H3K9Me3 modification needs more investigations as we didn’t find a clear increase in tumor tissue. Furthermore this epigenetic marks was already described in invasive regions of colorectal cancer tissues^30^. Based on results obtained in this study, we hypothesize that these two marks could come from either the environment of the tumor or from an inflammatory process associated with the tumor development. Markers produced by tumor cells (tumor-derived) or, for example, infiltrating leukocytes (tumor-associated) are both of interest from an early detection and/or diagnostic perspective as their levels could increase in the presence of disease, even at early stage.

Abnormal histone H3 methylation has been described to contribute to tumorigenesis in several types of cancers^31^. The increase of H3K27Me3 and its corresponding histone-modifying enzymes EZH2, have been described in tumor tissues from CRC patients relative to their normal counterpart, and have been associated with better prognosis^32^. H3K27Me3 levels in tissue have also been associated with CRC resistance to chemotherapeutics^33^. Karczmarski et al., have identified both H3K27Me3 and H3K27Ac as modifications upregulated in CRC tissues compared to healthy mucosa samples^34^. While most of these studies were performed on tissues, we showed in this present study that the elevation of these H3K27 marks is also observed in the blood of CRC patients by mass spectrometry and also by immunoassays. Loss of H4K20Me3 along with a reduction of H4K16Ac was observed across various cancer cell lines and tissue types^35^. In this study, we did not observe a significant change in the level of H4K20Me3 in tissue or in blood from CRC patients. This apparent discrepancy in tissue may be due to the limited number of samples analyzed. Nevertheless, we observed in tumor tissues an increase level of H4K20Me1 and Me2. We assume that this increase could partially reflect a loss of trimethylation on H4K20 residue as also suggested by the decrease of percentage of relative abundance of H4K20Me3 in tumor tissues samples compared to normal tissue (data not shown).

Acetylation of lysine residues is also a major histone modification involved in the regulation of chromatin structure and transcription. In this current study, we were not able to assess precisely the acetylation pattern of H4 in details. This fact is due to technical limitations of sample preparation and subsequent MS/MS acquisition for the given peptide. In contrast, we found an increased level of H3 acetylation in blood from CRC patients but not in the tissue tumor-normal pairs by mass spectrometry. This increase in CRC plasma samples was confirmed by immunoassay on the 9 CRC samples studied by both Nu.Q Capture-MS and immunoassay but was not statistically significant in the large clinical cohort study.

Immunoassays are commonly used to evaluate the levels of circulating biomarkers for diagnosis, prognosis or treatment follow-up. Nevertheless, we observed an apparent discrepancy between Nu.Q Capture-MS and immunoassays results as we confirmed in a large clinical cohort study, the increased levels of circulating H3.1-nucleosomes, H3K27Me2/3- and H3K9Me3-nucleosomes but not the elevated levels of H3K14Ac and H3K27Ac circulating nucleosomes. This observation could be mitigated by (1) the fact that the samples were tested with two different methods and (2) a larger sample size cohort were analyzed: (3) from a different geographical location. Indeed, mass spectrometry is well recognized as an analytical technique that allows an unbiased and quantitative evaluation of proteins in comparison of antibody-based methods that present some limitations linked to the use of reliable antibody^36^. Furthermore, in this present study, we were particularly interested to describe global level of circulating nucleosomes and their specific histone PTMs as potential blood biomarker rather than evaluating an underlying mechanism of up or down-regulation of a specific histone PTMs, therefore we have not normalized to total protein level. It would be useful to normalize both methods, ideally in a same way, to study relative up and down regulation and the histone modifiers regulating the epigenetic marks. Nonetheless, the method developed here will allow the identification of previously unknown candidates as potential biomarkers and drive the understanding of the epigenetic dysregulation in cancer.

To the best of our knowledge, aberrant H2A1R3 citrullination in cancer, and especially in CRC, has not been reported to date. Nevertheless, this finding is in line with the fact that PADI4, the enzyme responsible for the conversion of peptidylarginine to citrulline, was described as highly expressed in various malignant tumor tissues as well as in blood of patients with certain malignant tumors^37^. The results of the present study showed that H2A1R3Cit levels are elevated in both tissue and blood from the same patients, suggesting that circulating H2A1R3Cit may have a tumor origin and could be a good biomarker candidate for CRC diagnosis. We are currently developing an immunoassay to confirm this result.

In the present work, we proposed a novel method to identify and quantify circulating histone PTMs in plasma from cancer patients based on a specific capture of circulating nucleosomes followed by LC-MS/MS analysis. This method could be an interesting approach for biomarker discovery and the presence of histone PTMs in plasma can be used for clinical purposes. Indeed, the quantification of circulating H3K27Me3 and H3K9Me3 nucleosomes could be used as a non-invasive diagnostic approach for CRC and triaging test to prioritize patients for colonoscopy, especially in countries with serious colonoscopy capacity constraints. Further clinical studies will be required to validate these observations and conduct to lead markers.

## Methods

### Statement

All experiments and methods were performed in accordance with relevant guidelines and regulations. The study was approved by the Ethic Committee of the NTU and performed under IRB-approved protocols (IRB number: IRB201707065RIND). All patients gave informed consent to participate in the study.

### K2-EDTA plasma and tissue samples

For the proof-of-concept study, EDTA-Plasma and fresh frozen tissue samples from Caucasian colorectal cancer patients and Caucasian healthy donors were obtained from Dx BioSamples LLC (San Diego, California, US). Blood has been processed and frozed within 4 h of blood draw. The process includes a centrifugation step for 10 min at 1500 × *g* at 4 °C/39°F. Fresh Frozen of normal Adjacent Tissues were colon origin. Collection was performed under IRB-approved protocols from consented donors (CR00181954) (Supplementary Table 6).

The clinical study included 256 patients referred for colonoscopy to the National Taiwan University (NTU) Hospital. Patients were classified into the CRC group or negative colonoscopy group (control) based on their colonoscopy reports.

### Nucleosome immunoprecipitation (Nu.Q Capture)

Monoclonal anti-histone H3.1 antibody (Belgian Volition SRL, Namur, Belgium) was chemically bound on M280 Tosylactivated magnetic particles (#14204; Life Technologies Europe BV; Oslo; Norway). Briefly, the capture antibody diluted in coupling buffer (Borate buffer 0.1 M, pH 9.5) was incubated overnight at 37 °C with the magnetic particle. Following this incubation, the coated beads were blocked for 1 h at 37 °C with a blocking buffer (PBS with 0.1% Tween 20 and 1% BSA, pH7.4) and washed 3 times with a wash buffer (PBS with 0.1% Tween 20 with 0.1% BSA, pH7.4). Then, the coated beads were applied to the nucleosome immunoprecipitation protocol.

To isolate circulating nucleosome, 900 µl of plasma sample were incubated with 1 mg of coated magnetic particles for 1 h, at room temperature, under agitation. Then, thanks a magnet, nucleosomes captured by the coated beads were isolated from the rest of plasma (here referred after as “supernatant”). Finally, the nucleosomes-beads complex was washed, under agitation, three times with a wash buffer (PBS with 0.05% of Tween 20) and four times with PBS1x to remove unspecific captured proteins and detergent, respectively. The captured circulating nucleosome is ready for the further proteomic analysis.

In parallel, the supernatant was kept for a quality control experiment of the immunoprecipitation protocol using the Nu.Q H3.1 ELISA (#53511; Active Motif; San Diego; California; US).

### Tissue acid extraction

Tissue samples from CRC tumors and normal adjacent tissues were homogenized in tissue extraction buffer × 4 v/v (buffer to tissue volume) composed of 60 mM KCl, 15 mM NaCl, 4 mM MgCl2, 15 mM HEPES pH 7.6, 0.5% Triton, 0.5 mM Dithiothreitol (DTT) and 1 tablet (for 20 ml of the buffer) of Roche protease inhibitors (EpiQMAx, GmbH, Planegg, Germany). Homogenization was performed by douncing the tissue 10 × in 1.5 ml reaction tubes, followed by 12 cycles (30 s ON/30 s OFF) of sonication in a Bioruptor Pico device (Diagenode SA, Belgium). Samples were then centrifuged at 13,000 rpm for 30 min at 4 °C. Basic proteins (i.e., histones and others) were extracted as previously described using 0.2 M H_2_SO_4_ overnight, precipitated with trichloroacetic acid for 2 h, centrifuged at 13.000 rpm for 30 min at 4 °C, and pellets were washed 4 times with 200 µl of ice-cold acetone^38^. Pellets were finally resuspended in × 1 (v/v) Laemmli buffer and loaded onto a 4–20% SDS gradient gel (Serva, Germany). Histones bands were cut and processed as described previously for the analysis by mass spectrometry^39^.

### Nu.Q H3.1 assay

The residual nucleosome concentration in plasma sample after Nu.Q Capture application were measured using the Nu.Q H3.1 ELISA, a sandwich-type immunoassay, (#53511; Active Motif; San Diego; California; US) according to the manufacturer’s instructions. Briefly, 20 µl of unprocessed plasma samples or supernatant left after immunoprecipitation were incubated in a 96-well microtiter plate coated with an anti-H3.1 histone monoclonal antibody for 2h30 at room temperature. After the wash step, the plate was incubated with the HRP-conjugated anti-nucleosome antibody. Following this second incubation, the plate was washed again and 3,3′,5,5′-tétraméthylbenzidine (TMB) was added as a colorimetric substrate for the revelation. The absorbance was measured at 450 nm with a system luminometer (Xmark Microtitre plate reader; Biorad; Temse—Belgium). All samples were analyzed in duplicate. The nucleosome concentrations were determined using a four-parameter logistic regression of a reference standard curve.

As control for the immunoprecipitation, we calculated the percentage of nucleosome depletion. For this purpose, the concentration of nucleosomes left in supernatant after Nu.Q Capture was subtracted from the nucleosome concentration initially present in the crude sample.

### Nu.Q Immunoassay for clinical study

Five nucleosome structures were measured using quantitative Nu.Q Immunoassays (Belgian Volition SRL, Isnes, Belgium) according to the manufacturer’s instructions. Briefly, these sandwich immunoassays are based on magnetic beads and chemiluminescence technology and were performed on the IDS-i10 automated immunoanalyzer system (Immunodiagnostic Systems Ltd (IDS), UK). 50 µL of K2-EDTA plasma were incubated with acridinium ester labeled anti-nucleosome antibody. After this incubation step the magnetic particle beads, coated with the corresponding monoclonal anti-histone modification capture antibody (i.e., anti-histone H3.1, anti-histone H3K9Me3, anti-histone H3K27Me2/3, anti-histone H3K14Ac, or anti-histone H3K27Ac, respectively), were added. Following this second incubation, sandwich complexes comprising the anti-nucleosome antibody, nucleosome, and magnetic particle were separated from the unbound nucleosomes using a magnet. After a wash step, trigger reagents were added, and the light emitted by the acridinium ester was measured and expressed in relative light unit (RLU) by the system luminometer. All samples were analyzed in duplicate. If the %CV between the RLU of the duplicate measurements was above 20%, the sample was repeated. Each immunoassay contains standard samples composed of a define quantity recombinant nucleosomes to be used for the standard curve. Nucleosome/histone modification concentrations were determined by interpolating the mean RLU value of each tested samples against the standard curve and is expressed in ng/ml. The standard curve is construct using a four-parameter logistic function curve fitting. As each individual assay has its own standard, direct comparisons of the amounts measured are not possible between the assays.

### Western blotting

After the Nu.Q Capture protocol, the complex of circulating nucleosomes bounded to magnetic particles were incubated 5 minutes at 95 °C with Laemmli buffer (#1610747; Biorad; Temse—Belgium) containing 10% of 2-mercaptoethanol (#M6250-100ML; MercK; Overijse—Belgium). Following this incubation, the magnetic beads are removed and the denaturated proteins mixture are separated through an acrylamide gel (4–20% SDS-PAGE; #4561094; Biorad; Temse—Belgium) by electrophoresis under reducing conditions (Tris/Glycine/SDS; #1610772; Biorad; Temse—Belgium). Proteins were either Coomassie blue like stained (InstantBlue Ultrafast protein Stain; #ISB1L-1L; Sigma-Aldrich; Overijse—Belgium) or transferred onto a polyvinylidine difluoride (PVDF) membranes using a semi-dry transfer system for 3 minutes up to 25 V (#1704272; Biorad; Temse—Belgium). The PVDF membranes was then washed three times and incubated overnight at + 4 °C with an anti-histone H3 antibody (2 µg/ml—#80201; Active Motif; San Diego; California; US) diluted in Tris Buffer Saline containing 1% of Casein (#1706435; Biorad; Temse—Belgium). After this first antibody incubation, the membrane was washed three times and then incubated with a detection reagent (VeriBlot antibody for IP Detection Reagent, #ab131366; Abcam; Cambridge, UK) for 1 h at room temperature. Finally, after three additional washes, the membrane was incubated with ECL substrate (#34076; Life Technologies Europe; Sint-Kwintens-LenniK—Belgium) and chemiluminescent signals were acquired with the Fusion-FX6 instrument and software (Vilber; Marne-la-Vallée; France).

### Liquid chromatography with tandem mass spectrometry (LC-MS/MS)

#### Sample preparation for histone modification analysis by LC-MS/MS

Nucleosomes bound to magnetic beads were acylated and then digested with trypsin to obtain a peptide mixture compatible with the mass spectrometry analysis of histone modifications (EpiQMAx GmbH, Planegg, Germany)^38^. Heavy amino acid-labeled histone H3 peptides were added during sample preparation to each sample. These synthetic histone peptides are used for normalization to eliminate bias caused by sample preparation or instrumentation. Next, desalted peptides were injected in an Ultimate 3000 RSLCnano system (Thermo-Fisher Scientific, San Jose, CA) and separated on a 15‐cm analytical column (75 μm ID with ReproSil‐Pur C18‐AQ 2.4 μm from Dr. Maisch) using a gradient from 4% B to 90% B (solvent A 0.1% FA in water, solvent B 80% ACN, 0.1% FA in water) over 90 min at a flow rate of 300 nl/min. The effluent from the HPLC was directly electro-sprayed into a Q Exactive HF mass spectrometer (Thermo Fisher Scientific, San Jose, CA). The mass spectrometer was operated in data‐dependent mode to automatically switch between full scan MS and MS/MS acquisition. Survey full scan MS spectra (from m/z 375 to 1600) were acquired with resolution R = 60,000 at m/z 400 (AGC target of 3 × 10^6^). The 10 most intense peptide ions with charge states between 2 and 5 were sequentially isolated to a target value of 1 × 10^5^ and fragmented at 27% normalized collision energy. Typical mass spectrometric conditions were as follows: spray voltage, 1.5 kV; no sheath and auxiliary gas flow; heated capillary temperature, 250 °C; and ion selection threshold, 33,000 counts.

#### LC-MS/MS data analysis and quantification of histone modifications.

Raw files were searched with the Skyline software (MacLean et al., 2010) against canonical histone peptides and their respective PTMs with a precursor mass tolerance of 5 ppm. The chromatogram boundaries of + 1, + 2, + 3 and + 4 charged peaks were validated and the *Total Area MS1* under the first 4 isotopomers was extracted and used for relative quantification and comparison between experimental groups.

The raw peptides intensities were normalized using the intensities of spiked-in corresponding heavy peptides for H3 derived peptides (37 different heavy standards, which cover unmodified, acetylations and methylations (mono-, di- and tri-) on the following H3 lysines: K4, K9, K14, K27, K36, K56, and K79). For the normalization of peptides without spiked-in heavy standards (e.g., from H2A/B and H4), the normalization was done using the overall intensity trend of the heavy standards. More precisely, if we consider the vector of heavy intensities V for a particular modified peptide, the normalization factors NF, are computed as NF = max(V)/V. Then, the normalized light intensities are obtained by NF*W, where W is the vector of raw light intensities. The log2 of these normalized intensities are used for subsequent comparisons between experimental groups. For peptides without heavy standards, the normalization factors are computed as NF = max(Ӯ)/Ӯ, where Ӯ is the sample wise means of all heavy standards in each sample”.

### Statistical analysis

Mass spectrometry data are represented as Log2 of intensity. *p*-value were calculated using a student’s t-test. The Box plots for the clinical studies were computed with IBM SPSS Statistics software, version 26. The nucleosome levels are given as median and interquartiles, and total ranges. Median comparisons between the groups were performed by Mann–Whitney test.

## Supplementary Information

Supplementary Information 1.

Supplementary Information 2.

Supplementary Information 3.

Supplementary Information 4.

Supplementary Information 5.

Supplementary Information 6.

Supplementary Information 7.

Supplementary Information 8.

Supplementary Information 9.

## References

[1] Bray F (2018). Global cancer statistics 2018: GLOBOCAN estimates of incidence and mortality worldwide for 36 cancers in 185 countries. CA Cancer J. Clin..

[2] Hong SN (2018). Genetic and epigenetic alterations of colorectal cancer. Intest. Res..

[3] Bardhan K, Liu K (2013). Epigenetics and colorectal cancer pathogenesis. Cancers (Basel).

[4] Portela A, Esteller M (2010). Epigenetic modifications and human disease. Nat. Biotechnol..

[5] Tessarz P, Kouzarides T (2014). Histone core modifications regulating nucleosome structure and dynamics. Nat. Rev. Mol. Cell Biol..

[6] Oliveri M (2001). DNase I mediates internucleosomal DNA degradation in human cells undergoing drug-induced apoptosis. Eur. J. Immunol..

[7] Elshimali YI, Khaddour H, Sarkissyan M, Wu Y, Vadgama JV (2013). The clinical utilization of circulating cell free DNA (CCFDNA) in blood of cancer patients. Int. J. Mol. Sci..

[8] Holdenrieder S (2008). Clinical relevance of circulating nucleosomes in cancer. Ann. N. Y. Acad. Sci..

[9] Rahier JF (2017). Circulating nucleosomes as new blood-based biomarkers for detection of colorectal cancer. Clin. Epigenetics.

[10] Schwarzenbach H, Hoon DS, Pantel K (2011). Cell-free nucleic acids as biomarkers in cancer patients. Nat. Rev. Cancer.

[11] Seligson DB (2009). Global levels of histone modifications predict prognosis in different cancers. Am. J. Pathol..

[12] Reis AH, Vargas FR, Lemos B (2016). Biomarkers of genome instability and cancer epigenetics. Tumour Biol..

[13] Fullgrabe J, Kavanagh E, Joseph B (2011). Histone onco-modifications. Oncogene.

[14] Gezer U (2015). Histone methylation marks on circulating nucleosomes as novel blood-based biomarker in colorectal cancer. Int. J. Mol. Sci..

[15] Holdenrieder S (2014). Novel serum nucleosomics biomarkers for the detection of colorectal cancer. Anticancer Res..

[16] Bauden M (2015). Circulating nucleosomes as epigenetic biomarkers in pancreatic cancer. Clin. Epigenetics.

[17] Berdasco M, Esteller M (2019). Clinical epigenetics: seizing opportunities for translation. Nat. Rev. Genet..

[18] Garcia-Gimenez JL (2017). A new mass spectrometry-based method for the quantification of histones in plasma from septic shock patients. Sci. Rep..

[19] Volker-Albert MC, Schmidt A, Barth TK, Forne I, Imhof A (1832). Detection of histone modification dynamics during the cell cycle by MS-based proteomics. Methods Mol. Biol..

[20] Washburn MP, Zhao Y, Garcia BA (2016). Reshaping the chromatin and epigenetic landscapes with quantitative mass spectrometry. Mol. Cell. Proteomics.

[21] Noberini R (2019). Profiling of epigenetic features in clinical samples reveals novel widespread changes in cancer. Cancers (Basel).

[22] Bannister AJ, Kouzarides T (2011). Regulation of chromatin by histone modifications. Cell Res..

[23] Khan SA, Reddy D, Gupta S (2015). Global histone post-translational modifications and cancer: biomarkers for diagnosis, prognosis and treatment?. World J. Biol. Chem..

[24] Zhang C (2016). Quantitative proteomic analysis of histone modifications in decitabine sensitive and resistant leukemia cell lines. Clin. Proteomics.

[25] Gruppuso PA (2018). Stability of histone post-translational modifications in samples derived from liver tissue and primary hepatic cells. PLoS ONE.

[26] An S (2020). Histone tail analysis reveals H3K36me2 and H4K16ac as epigenetic signatures of diffuse intrinsic pontine glioma. J. Exp. Clin. Cancer Res..

[27] Rasmussen L (2018). Circulating cell-free nucleosomes as biomarkers for early detection of colorectal cancer. Oncotarget.

[28] Voigt P (2012). Asymmetrically modified nucleosomes. Cell.

[29] Silveira AB (2019). H3.3 K27M depletion increases differentiation and extends latency of diffuse intrinsic pontine glioma growth in vivo. Acta Neuropathol..

[30] Yokoyama, Y. *et al.* Cancer-associated upregulation of histone H3 lysine 9 trimethylation promotes cell motility in vitro and drives tumor formation in vivo. *Cancer Sci.* **104**(7), 889–895 (2013). 10.1111/cas.12166 PMC7657232 23557258

[31] Greer EL, Shi Y (2012). Histone methylation: a dynamic mark in health, disease and inheritance. Nat. Rev. Genet..

[32] Benard A (2014). Prognostic value of polycomb proteins EZH2, BMI1 and SUZ12 and histone modification H3K27me3 in colorectal cancer. PLoS ONE.

[33] Wang Q (2020). Elevating H3K27me3 level sensitizes colorectal cancer to oxaliplatin. J. Mol. Cell Biol..

[34] Karczmarski J (2014). Histone H3 lysine 27 acetylation is altered in colon cancer. Clin. Proteomics.

[35] Fraga MF (2005). Loss of acetylation at Lys16 and trimethylation at Lys20 of histone H4 is a common hallmark of human cancer. Nat. Genet..

[36] Noberini R, Robusti G, Bonaldi T (2021). Mass spectrometry-based characterization of histones in clinical samples: applications, progresses, and challenges. FEBS J..

[37] Chang X (2009). Increased PADI4 expression in blood and tissues of patients with malignant tumors. BMC Cancer.

[38] Volker-Albert MC, Schmidt A, Forne I, Imhof A (2018). Analysis of histone modifications by mass spectrometry. Curr. Protoc. Protein Sci..

[39] Villar-Garea A, Israel L, Imhof A (2008). Analysis of histone modifications by mass spectrometry. Curr. Protoc. Protein Sci..

