# Supplementary material for: A novel proteomics approach to epigenetic profiling of circulating nucleosomes
Source: Sci Rep. 2021 Mar 31;11:7256. doi: 10.1038/s41598-021-86630-3 (PMC8012598; doi:10.1038/s41598-021-86630-3)
Supplement: Supplementary file 3 — Supplementary Information 3. [file 41598_2021_86630_MOESM3_ESM.docx]

**Supplementary Table 3: Volcanoplot details.**

1. **Volcano plot: Plasma samples**

| **Label** | **Modification** | **P-value** | **Change** |
| --- | --- | --- | --- |
| 1 | H3.3_K27me3 | <0,001 | Down |
| 2 | H3.3__K36ac | <0,001 | Up |
| 3 | H3_K4 | 0,001 | Up |
| 4 | H3.1_K27me2 | 0,002 | Up |
| 5 | H3_K9me1 | 0,003 | Up |
| 6 | H2A1_GKQGGKAR | 0,003 | Up |
| 7 | H3_K9me2 | 0,003 | Up |
| 8 | H3.1_K27ac | 0,004 | Up |
| 9 | H3_K9me3 | 0,004 | Up |
| 10 | H2A1_AGLQFPVGR | 0,005 | Up |
| 11 | H3.3_K27_K36 | 0,01 | Up |
| 12 | H3_YRPGTVALR | 0,01 | Up |
| 13 | H2A1_R3_cit | 0,01 | Up |
| 14 | H3_K56 | 0,02 | Up |
| 15 | H3.1_K27me1_K36me3 | 0,02 | Down |
| 16 | H3_K18ac_K23ac | 0,03 | Up |
| 17 | H3_K9_K14 | 0,03 | Up |
| 18 | H3_K23ac | 0,03 | Up |
| 19 | H4_4..17 | 0,03 | Up |
| 20 | H3_K14ac | 0,04 | Up |
| 21 | H3_K18_K23 | 0,04 | Up |
| 22 | H3_K79 | 0,04 | Up |
| 23 | H4_DNIQGITKPAIR | 0,05 | Up |

1. **Volcano plot: Tissue samples**

| **Label** | **Modification** | **Pvalue** | **Change** |
| --- | --- | --- | --- |
| 1 | H2A1_R3_cit | 0,001 | Up |
| 2 | H2A1_AGLQFPVGR | 0,002 | Up |
| 3 | H3_K79 | 0,002 | Up |
| 4 | H4_K20 | 0,003 | Up |
| 5 | H2A1_GKQGGKAR | 0,003 | Up |
| 6 | H3.1_K27me2 | 0,004 | Up |
| 7 | H3_K56me2 | 0,01 | Up |
| 8 | H3.3_K27me3_K36me2 | 0,01 | Down |
| 9 | H3.1_K27me3 | 0,02 | Up |
| 10 | H3.1_K27ac | 0,02 | Up |
| 11 | H3.3_K27me3_K36me1 | 0,02 | Down |
| 12 | H3.1_K27me3_K36me2 | 0,03 | Up |
| 13 | H3.1_K27me3_K36me3 | 0,03 | Up |
| 14 | H3.1_K27me3_K36me1 | 0,03 | Up |
| 15 | H4_4..17_3ac | 0,03 | Up |
| 16 | H3.1_K27me2_K36me2 | 0,03 | Up |
| 17 | H3.3_K27me3_K36me3 | 0,03 | Down |
| 18 | H4_K20me2 | 0,04 | Up |
| 19 | H4_DNIQGITKPAIR | 0,04 | Up |
| 20 | H4_4..17_4ac | 0,04 | Up |
| 21 | H4_4..17_2ac | 0,04 | Up |
| 22 | H3.1_K27me1_K36me3 | 0,04 | Up |
| 23 | H3.1_K27me1_K36me2 | 0,04 | Up |
| 24 | H4_K20me1 | 0,05 | Up |
| 25 | H3.1_K27me2_K36me1 | 0,05 | Up |
| 26 | H3.3_K27me2_K36me1 | 0,05 | Down |
