# Supplementary material for: A novel proteomics approach to epigenetic profiling of circulating nucleosomes
Source: Sci Rep. 2021 Mar 31;11:7256. doi: 10.1038/s41598-021-86630-3 (PMC8012598; doi:10.1038/s41598-021-86630-3)
Supplement: Supplementary file 5 — Supplementary Information 5. [file 41598_2021_86630_MOESM5_ESM.docx]

**Supplementary Table 5: Venn Diagram details list.**

| **Tissue Only** | | **Common** | | **Plasma Only** | |
| --- | --- | --- | --- | --- | --- |
| **modifications**  **(p<0.05)** | **CRC vs NAT** | **modifications**  **(p<0.05)** | **CRC vs NAT/ healthy donors** | **modifications**  **(p<0.05)** | **CRC vs healthy donors** |
| H3.1_K27Me3 | up | H2A1_R3Cit | up | H3_K14Ac | up |
| H4_4...17_3Ac | up | H3.1_K27Ac | up | H3_K23Ac | up |
| H3.3_K27Me3_K36Me3 | down | H3.1_K27Me2 | up | H3.3_K27Me3 | down |
| H3.1_K27Me1_K36Me2 | up |  |  | H3_K9Me3 | up |
| H4_K20Me1 | up |  |  | H3_K9Me2 | up |
| H4_4...17_2Ac | up |  |  | H3_K9Me1 | up |
| H4_K20Me2 | up |  |  | H3_K18Ac_K23Ac | up |
| H3.1_K27Me2_K36Me2 | up |  |  | H3.3_K36Ac | up |
| H3.3_K27Me2_K36Me1 | down |  |  | H3.1_K27Me1_K36Me3 | up |
| H3.1_K27Me3_K36Me2 | up |  |  |  |  |
| H3.1_K27Me3_K36Me3 | up |  |  |  |  |
| H3.3_K27Me3_K36Me2 | down |  |  |  |  |
| H3.1_K27Me3_K36Me1 | up |  |  |  |  |
| H3.1_K27Me2_K36Me1 | up |  |  |  |  |
| H3.3_K27Me3_K36Me1 | down |  |  |  |  |
| H3_K56Me2 | up |  |  |  |  |
| H4_4…17_4Ac | up |  |  |  |  |
| H3.1_K27Me1_K36Me3 | up |  |  |  |  |
