# Supplementary material for: A novel proteomics approach to epigenetic profiling of circulating nucleosomes
Source: Sci Rep. 2021 Mar 31;11:7256. doi: 10.1038/s41598-021-86630-3 (PMC8012598; doi:10.1038/s41598-021-86630-3)
Supplement: Supplementary file 6 — Supplementary Information 6. [file 41598_2021_86630_MOESM6_ESM.docx]

**Supplementary Table 6: Clinical information of healthy donors (n=9) and paired plasma, tumor and normal adjacent tissue (n=9).**

| Sample | Sex | Age | Histological diagnosis | TNM | Tumor content (%) |
| --- | --- | --- | --- | --- | --- |
| #1 | M | 68 | adenocarcinoma  (Ascending colon) | T3N1aM0 | 100 |
| #2 | M | 40 | adenocarcinoma  (Sigmoid colon) | T4bN2bM1c | 100 |
| #3 | F | 64 | adenocarcinoma  (Sigmoid colon) | T3N0M0 | 80 |
| #4 | F | 66 | adenocarcinoma  (Ascending colon) | T3N0M0 | 100 |
| #5 | F | 65 | adenocarcinoma  (Sigmoid colon) | T2N1cM0 | 100 |
| #6 | M | 73 | adenocarcinoma  (Sigmoid colon) | T3N1bM0 | 90 |
| #7 | M | 64 | adenocarcinoma  (Sigmoid colon) | T3N1aM0 | 90 |
| #8 | F | 62 | adenocarcinoma  (Ascending colon) | T3N2aM0 | 70 |
| #9 | F | 68 | adenocarcinoma  (Sigmoid colon) | T3N1bM0 | 90 |
| #1 | M | 66 | healthy donor | - | - |
| #2 | F | 61 | healthy donor | - | - |
| #3 | F | 68 | healthy donor | - | - |
| #4 | F | 69 | healthy donor | - | - |
| #5 | F | 71 | healthy donor | - | - |
| #6 | F | 74 | healthy donor | - | - |
| #7 | F | 65 | healthy donor | - | - |
| #8 | F | 69 | healthy donor | - | - |
| #9 | F | 73 | healthy donor | - | - |
