# Supplementary figures and images for: A novel proteomics approach to epigenetic profiling of circulating nucleosomes
Source: Sci Rep. 2021 Mar 31;11:7256. doi: 10.1038/s41598-021-86630-3 (PMC8012598; doi:10.1038/s41598-021-86630-3)

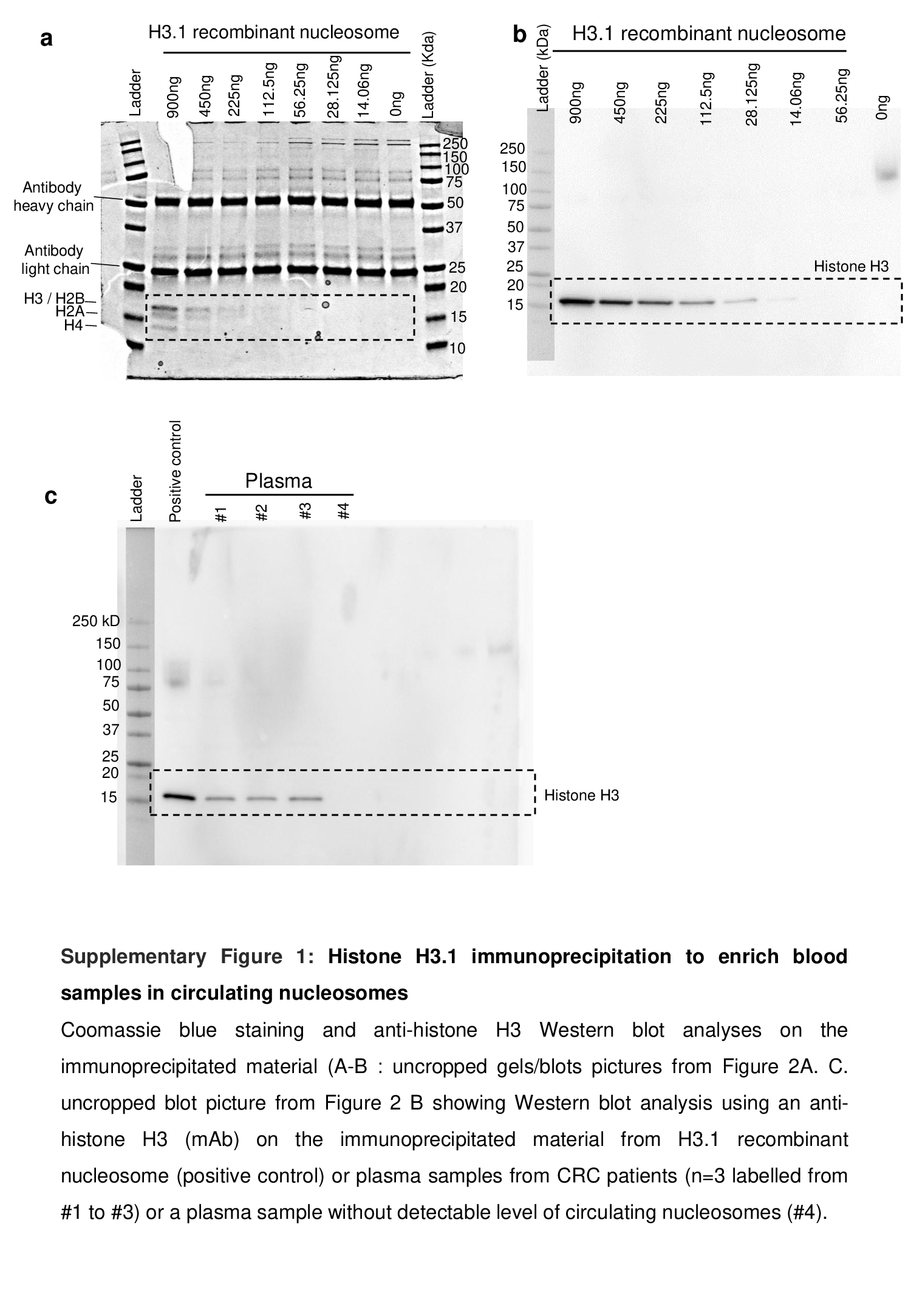

Supplement: Supplementary file 7 — Supplementary Information 7. [file 41598_2021_86630_MOESM7_ESM.jpeg]

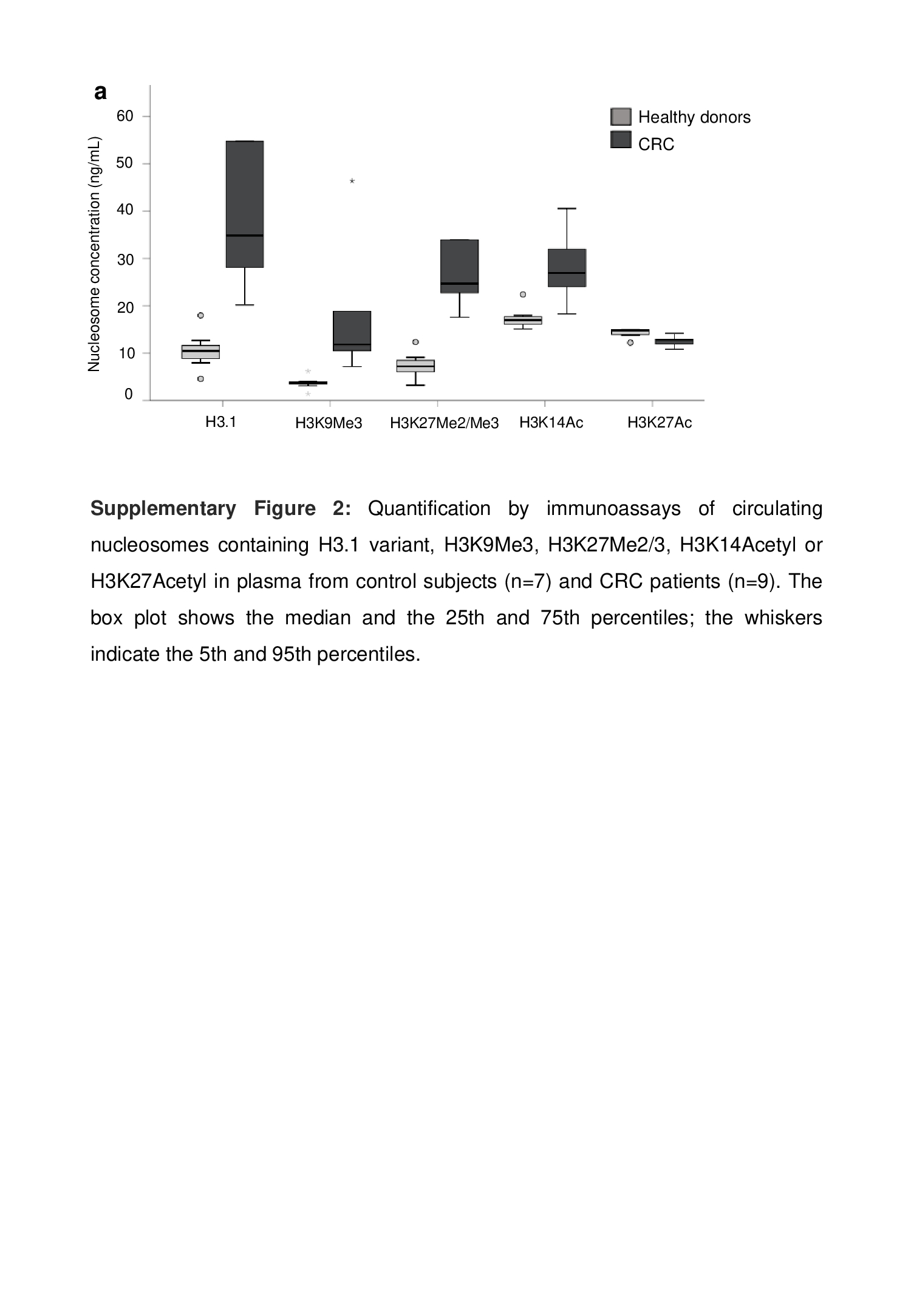

Supplement: Supplementary file 8 — Supplementary Information 8. [file 41598_2021_86630_MOESM8_ESM.jpeg]
