# Supplementary material for: A novel proteomics approach to epigenetic profiling of circulating nucleosomes
Source: Sci Rep. 2021 Mar 31;11:7256. doi: 10.1038/s41598-021-86630-3 (PMC8012598; doi:10.1038/s41598-021-86630-3)
Supplement: Supplementary file 9 — Supplementary Information 9. [file 41598_2021_86630_MOESM9_ESM.docx]

**Supplementary Figure 1: Histone H3.1 immunoprecipitation to enrich blood samples in circulating nucleosomes**

Coomassie blue staining and anti-histone H3 Western blot analyses on the immunoprecipitated material (A-B : uncropped gels/blots pictures from Figure 2A. C. uncropped blot picture from Figure 2 B showing Western blot analysis using an anti-histone H3 (mAb) on the immunoprecipitated material from H3.1 recombinant nucleosome (positive control) or plasma samples from CRC patients (n=3 labelled from #1 to #3) or a plasma sample without detectable level of circulating nucleosomes (#4).

**Supplementary Figure 2:** Quantification by immunoassays of circulating nucleosomes containing H3.1 variant, H3K9Me3, H3K27Me2/3, H3K14Acetyl or H3K27Acetyl in plasma from control subjects (n=7) and CRC patients (n=9). The box plot shows the median and the 25th and 75th percentiles; the whiskers indicate the 5th and 95th percentiles.

**Supplementary Table 1: Proteoforms detected in plasma samples and on immunoprecipitated materials.** Abundance here refers to the average MS intensity of all proteotypic peptides of the proteins represented in the plot. Indeed, the average intensity of the 2 technical replicates in each experimental group was calculated peptide-sequence-wise. Next, the average intensity of all peptides above was summarized protein-ID-wise. After this, average intensity values were log2-transformed and plotted as a heatmap.

**Supplementary Table 2: Histone proteoforms detected in plasma samples.**

This table lists the histone proteoforms identified by the Nu.Q Capture- MS analysis in plasma samples. The mean of log 2 intensity value for each PTM from healthy donors or CRC patients are recorded. p-values were determined by student’s t- Test analysis (n=9 healthy donors and n = 9 CRC samples).

**Supplementary Table 3:** **Volcanoplot details A.** This table lists the histone proteoforms showing a significant fold change in plasma of CRC patients vs healthy donors. **B**. This table lists the histone proteoforms significantly showing a significant fold change in tumor tissue in comparison to normal adjacent tissue.

**Supplementary Table 4: Histone proteoforms detected in tumor and Normal adjacent tissues.**

This table lists the list of histone proteoforms identified in tumor and Normal adjacent tissues. The mean of log 2 intensity value for each PTM from normal or tumor tissues are recorded. p-values were determined by student’s t-Test analysis (n=9 paired samples).

**Supplementary Table 5: Venn Diagram details list.**

This table shows the list of PTMs identified as possessing statistically significant differential expression between CRC tissue and NAT tissues (n= 9 paired samples) or between healthy donors (n=9) and CRC plasma samples (n=9) (p ≤0.05).

**Supplementary Table 6: Clinical information of healthy donors (n=9) and paired plasma, tumor and normal adjacent tissue (n=9).**

TNM : tumor (T), nodes (N), and metastases (M) classification.
